# Microscopic Image Segmentation and Morphological Characterization of Novel Chitosan/Silica Nanoparticle/Nisin Films Using Antimicrobial Technique for Blueberry Preservation

**DOI:** 10.3390/membranes11050303

**Published:** 2021-04-21

**Authors:** Rokayya Sami, Schahrazad Soltane, Mahmoud Helal

**Affiliations:** 1 Department of Food Science and Nutrition, College of Sciences, Taif University, P.O. Box 11099, Taif 21944, Saudi Arabia; 2 Department of Computer Engineering, Faculty of Computers and Information Technology, Taif University, P.O. Box 11099, Taif 21944, Saudi Arabia; shasoltane@tu.edu.sa; 3 Department of Mechanical Engineering, Faculty of Engineering, Taif University, P.O. Box 11099, Taif 21944, Saudi Arabia; mo.helal@tu.edu.sa

**Keywords:** image segmentation, image classification, *K*-means clustering, Sobel, novel films, nanoparticle, preservation

## Abstract

In the current work, the characterization of novel chitosan/silica nanoparticle/nisin films with the addition of nisin as an antimicrobial technique for blueberry preservation during storage is investigated. Chitosan/Silica Nanoparticle/N (CH-SN-N) films presented a stable suspension as the surface loads (45.9 mV) and the distribution was considered broad (0.62). The result shows that the pH value was increased gradually with the addition of nisin to 4.12, while the turbidity was the highest at 0.39. The content of the insoluble matter and contact angle were the highest for the Chitosan/Silica Nanoparticle (CH-SN) film at 5.68%. The use of nano-materials in chitosan films decreased the material ductility, reduced the tensile strength and elongation-at-break of the membrane. The coated blueberries with Chitosan/Silica Nanoparticle/N films reported the lowest microbial contamination counts at 2.82 log CFU/g followed by Chitosan/Silica Nanoparticle at 3.73 and 3.58 log CFU/g for the aerobic bacteria, molds, and yeasts population, respectively. It was observed that (CH) film extracted 94 regions with an average size of 449.10, at the same time (CH-SN) film extracted 169 regions with an average size of 130.53. The (CH-SN-N) film presented the best result at 5.19%. It could be observed that the size of the total region of the fruit for the (CH) case was the smallest (1663 pixels), which implied that the fruit lost moisture content. As a conclusion, (CH-SN-N) film is recommended for blueberry preservation to prolong the shelf-life during storage.

## 1. Introduction

Silicon dioxide (SiO_2_) is well known as ultra-thin films that are efficiently used for modern nanotechnologies techniques such as surface passivation materials, catalysts, anti-fogging, dielectric materials, self-cleaning, and anti-corrosion as it considered an environmental and friendly nature component [1,2]. Moreover, the Si-SiO_2_ system in powder or thin-film form is widely used in the food industry and food preservation fields as nanoparticle functions can act as a property enhancer, which is the most abundant material in the earth’s crust [3,4]. The SiO_2_ chemical formula is structurally similar to diamond, safe and non-toxic, and is found to be crystalline with a white color [5]. Moreover, the whiteness of nano-silicon dioxide could be colored with all suitable colors to fit all food products [6]. Improvements in physical and mechanical properties, such as hardness, high porosity, low index of refraction, and thermal conductivity, could be achieved with nanomaterials [7,8].

Recently, a great interest has arisen for such nanocomposites active materials in the nanoscale from consumers and productions for stable environmental products. Silica nanoparticle films are inexpensive, with a small content of chemical impurities, taken into consideration as proper techniques for preservative coatings when compared to the common composite materials [9,10]. Nanocomposite SiO_2_ can preserve the freshness of foods and inhibit rancidity, color change, nutrient loss, dehydration, sticking, microbial attack *in-vitro*, and *in-vivo* [11]. Hence, it can maintain respiration rates, acceptable levels of sensory evaluations, physiological qualities, and longer shelf-life during storage [12]. 

Chitosan films are commonly used in food packaging and coating materials prepared by the solvent casting with dilute aqueous acid solutions. Chitosan films achieved high mechanical properties in stress tests due to the porosity of the membranes [13]. Chitosan improves the gas barrier and the mechanical properties by the interaction between chitosan chains and the chemical components such as oil, fatty acids, and polysaccharides [14,15,16,17]. Nisin is considered a non-toxic food additive that acts as an active anti-microbial agent for various applications of foods [18]. Image processing, especially image segmentation, is commonly used by extracting the regions and their characteristics [19,20]. Researchers realized that images are often drowned in useless information such as noise and information redundancy [21]. The fundamental process in visual recognition is to decrease this quantity of information by keeping only the relevant elements. Many research works have been developed to reduce noise, edge detection, and region extraction methods by focusing on the relevant information contain [22,23]. Studies have shown that the human operator can recognize the object with a simple observation of its edges. The main information is in the edge and regions of the image. The technique to extract that information and reduce their quantities is called segmentation. An important goal of image segmentation is to separate the object and background. For several years, segmentation has been the subject of several types of research to reduce the complexity of images by a simple description [24,25]. However, there is no generalized method for a large variety of images. Several techniques exist, each one with a specific domain of application [24]. Several segmentation techniques are based on edge detection, region growing, adaptive filtering, mathematical morphology, watershed clustering, and semantic segmentation. Zheng et al. [26] proposed an adaptive *K*-means image segmentation generating accurate segmentation results with simple operation, avoiding the interactive input of *K* value. The method transforms the color space of images into LAB color space. In medical research, some general segmentation methods have found applications in biomedical image classification, especially in blood cell image processing [27]. Lin et al. [28] proposed an improved algorithm based on the feature weight adaptive *K*-means clustering to extract diseased cells. Clustering methods have been used to segment the magnetic resonance imaging (RMI) brain images [29]. The method combines *K*-means and fuzzy c-means clustering algorithms (fuzzy *k*-c-means clustering). Khashman et al. [30] proposed the use of morphological analysis of microscopic images (leukemia) to identify the disease and segment the infected cell images. The process of segmentation was included two enhanced images for each cell: the cytoplasm and the nuclei regions. In the process of identification, features were extracted from the leukemia cells. Soltane [20] used an adaptive method to segment the image using a mathematic function called variogram in order to guide the optimal edge orientation and extract edges.

The current study aims to investigate the characterization of novel chitosan/silica nanoparticle films with the addition of nisin as an antimicrobial technique for blueberry preservation during storage. The characterization includes morphological properties, color, ζ-potential, particle size distribution diameters, polydispersity index, acidity, turbidity, solubility in water, contact angle, and mechanical tensile strength with the addition of the microbial contamination analysis of coated blueberries during storage. The current segmentation approach aims to extract the regions from the microscopic images of three categories (Chitosan (CH), Chitosan/Silica Nanoparticle (CH-SN), and Chitosan/Silica Nanoparticle/N (CH-SN-N) to determine their morphological characteristics such as a number of areas, areas size, perimeter, and area grayscale to compare the three categories of images. The image processing study is enforced by the chemical–physical characteristics of the films. In the image processing part, the approach involves three levels: preprocessing, segmentation, and post-processing. The proposed automatic method is adapted to detect the holes in the microscopic images and segment the component nuclei and define some characteristics such as form, size, color by the *K*-means method to classify the different image regions and separate the holes from the background.

## 2. Materials and Methods

### 2.1. Materials

Chitosan derived from crab shell (>85% deacetylation) and nanosilicon dioxide (25 nm) waspurchased from Sigma Chemical Co. (St. Louis, MO, USA). Glycerol and glacial acetic acid were local products of analytical grade. 

#### 2.1.1. Films Preparation and Production

Chitosan powder (1%) was solubilized in deionized water with acetic acid (1%) and glycerol (0.5%) under continuous stirring until the complete dissolution. Silica Nanoparticle (1%) (Chitosan/Silica Nanoparticle) was dispersed in chitosan then sonicated (KQ-250 E, China) for 30 min to ensure the homogenization was completed, while nisin (1%) is blended with (Chitosan/Silica Nanoparticle) to prepare (Chitosan/Silica Nanoparticle/N). Some of the solutions were spread on Petri dishes and settled with a portion (30 g) to ensure a constant thickness for the film yield. Petri dishes were freeze-dried (ALPHA 1-4 LSC, Osterode am Harz, Germany) at −50 °C and 0.04 amber for 48 h. Dried films were removed and stored at 27 °C and 65% relative humidity until the characterization processes. The other coating solutions were applied on blueberry fruits to prolong the shelf-life during storage at commercial temperature.

#### 2.1.2. Sample Treatments

Fresh blueberries in uniform size and damage-free were transmitted to the Department of Food Science, Taif City, Saudi Arabia. Blueberries were divided into four groups; Control samples were dipped into deionized water, while the other groups were dipped into coating solutions such as Chitosan, Chitosan/Silica Nanoparticle, and Chitosan/Silica Nanoparticle/N for 15 min and then air-dried. All the physicochemical characterize and microbial contamination were evaluated at an interval of three days and carried out up to nine days during storage.

#### 2.1.3. Determination of Morphological Properties

Liner dimensions of films such as length (L, mm) and width (W, mm) and were evaluated by a dial-micrometer (Mitutoyo Manufacturing, Tokyo, Japan) with a sensitivity of 0.01 mm. The mass (M, g) was recorded by an electric sensitive balance (AUY220 Shimadzu, analytical scale, Harbin, China) with an accuracy of ±0.01 g [31,32,33,34,35]. Film thickness (T, mm) was evaluated with a dial-micrometer at 10 random points and the average value was obtained [36].

#### 2.1.4. Determination of Film Color 

The color parameters were evaluated by using a ZE-6000 color meter (Nippon Denshoku Co., Tokyo, Japan). The parameters (L*, a*, and b*) of the films were expressed as L* (lightness), a* (red-green), and b* (yellow-blue) values [37,38].

#### 2.1.5. Determination of ζ-Potential, Particle Size Distribution, and Polydispersity Index

The diameters of ζ-potential (mV) and particle size distribution (nm) were evaluated by using Zetasizer Nano-ZS90 (Mastersizer 2000; Malvern Instruments, West Midlands, Worcestershire, UK) with a Hydro 2000MU (A) wet liquid feeder (λ = 633 nm) and a 90° angle. Films were dispersed in water at 0.04 wt% to evaluate Dz and the polydispersity index, while ζ-potential measurement was diluted at 0.08 wt% [39].

#### 2.1.6. Determination of Acidity and Turbidity

The acidity (pH) reading was directly examined by a digital pH meter (MP 220, Metler Toledo, Greifensee, Switzerland) of film solutions [40]. The turbidity of the films was diluted 50 times in PBS (0.01 M, pH 7.0) as a reference and evaluated at 600 nm with a UV-2550 ultraviolet-visible spectrophotometer (Shimadzu Co., Shanghai, China) [39].

#### 2.1.7. Determination of Solubility in Water and Contact Angle

The solubility of the films in water was evaluated according to the method by Lianos et al. [13]. The film samples were cut into (2 × 6 cm) and were stored in a desiccator with P2O5 (0% RH) for 72 h. Dry films were weighed to the nearest 0.01 g, immersed in phosphate buffer solutions with respective (pH = 5.9), and stirred for 1 h at the ambient temperature. The contact angle (degree) was measured by using a colored water droplet with a digital microscope camera (U-VISION MV500, China). The reported date was an average of eight measurements [41,42].

#### 2.1.8. Determination of Mechanical Tensile Strength Tests

The mechanical tensile strength tests were performed using a texture analyzer (TA-XT, Stable Micro Systems, Surrey, UK) with the Accurate Magnetic Thickness Gauge (AMTG) probe. An initial grip separation of 30 mm and 10 mm/s speed were used. Test strip dimensions were (2 × 6 cm) with a repetition of eight measurements per film [36,43]. The films were stretched to failure, generating a modulus of elasticity (E, MPa), stress (σ, MPa), breaking force (FB, N), fracture stress (σF, MPa), extensibility (mm), and strain at break (εB, mm/mm).

#### 2.1.9. Determination of Microbial Contamination Analysis

The analyses of microbial contamination such as aerobic bacteria, molds, and yeasts counts were evaluated at an interval of three days and carried out up to nine days during storage according to the methodology described by Bambace et al. [44]. Aerobic bacteria, molds, and yeasts were performed using a rose bengal medium (GB4789.15-2016) and (GB4789.2-2016), respectively. All the plates were incubated at (±27 °C) for 3–5 days. At the end of the incubation period, the microbial colonies were expressed as log CFU/g (colony forming units) per gram from four sample containers and three replicate counts for each container.

#### 2.1.10. Microscopic Images Dataset

**Figure 1:**
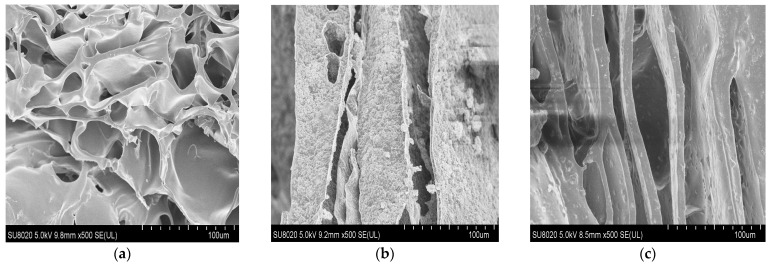
Scanning electron microscopy (SEM) images surfaces; Chitosan film (**a**), Chitosan/Silica Nanoparticle film (**b**), and Chitosan/Silica Nanoparticle/N film (**c**).

Images of the dataset were acquired with a Hitachi 8020 (Tokyo, Japan) scanning electron microscopy (SEM). Three classes of images were acquired with an optical zoom of 100 um and a resolution of 1280 × 960 pixels. Image samples for Chitosan, Chitosan/Silica Nanoparticle, and Chitosan/Silica Nanoparticle/N are presented in Figure 1.

### 2.2. Microscopic Image Segmentation Methodology

#### 2.2.1. Image Processing Steps

**Figure 2:**
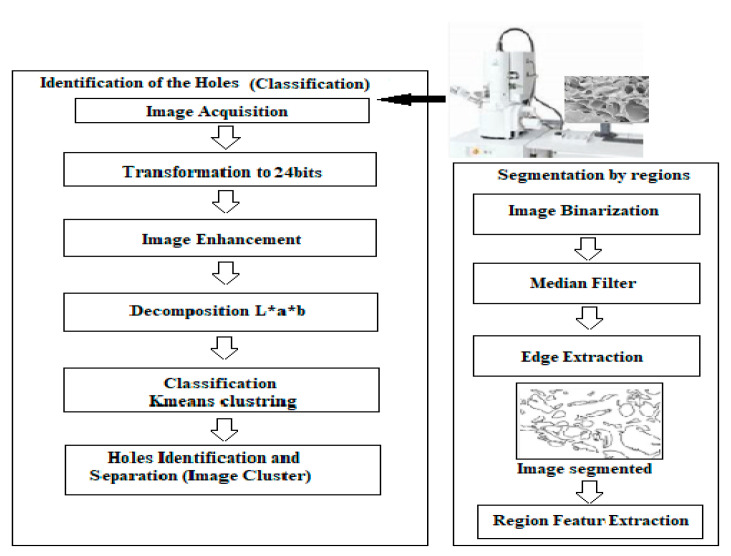
The proposed flow chart diagram of the image processing steps.

The main aim was to automatically segment the images using the *K*-means clustering to extract the holes and segment the image. The entire process is summarized as in Figure 2.

**Step 1:** Image acquisition: Acquisition image of the scanning electron microscopy (SEM) images in grayscale 8 bits and resolution of 1280 × 960 pixels.

**Step 2:** Image transformation to 24 bits (RGB color space).

**Step 3:** Image enhancements: The image was normalized by changing the range of pixel intensity values to be in a range of (0 and 1). The extreme pixels are removed, and the image is scaled between 0 and 1.

**Step 4:** Transformation: The image was transformed to L*a*b* color space. The algorithm converted the image to CIE L*a*b* color space to quantify the visual differences. The L*a*b* color spaces were derived from the CIE XYZ values which consisted of a luminosity layer ‘L*’, chromaticity-layer ‘a*’, and chromaticity-layer ‘b*’. The algorithm measured the difference between the two color spaces by using the Euclidean distance metric.

**Step 5:** Classification: Classify the image in ‘L*a*’ space by using *K*-means clustering algorithm and separate object groups. The *K*-means clustering algorithm treats each object as a location in space. It finds partitions such as objects within each cluster are as close to each other as possible and as far from objects in other clusters as possible. Euclidean similarity distance metric method was used to separate the holes from the background.

**Step 6:** Labeling: The algorithm labeled the image pixels by using *K*-means results and generated an index corresponding to the cluster.

**Step 7:** Using the pixel labels, the algorithm separated objects by color, created a new classified image, and three different cluster images.

**Step 8:** Using the median filter on cluster images (holes) to eliminate the small region and noise (impulse).

**Step 9:** Segmentation of holes by regions. 

**Step 10:** Characterization: From each region, the morphological characteristics (total pixel areas, mean areas size, perimeter, grayscale,…) were extracted to compare the different image classes.

The proposed approach used the *K*-means method to classify the different image regions, separate the holes from the background in the various film cases. The performance of the system consists of the automatic choice of the cluster parameter using the Elbow method.

#### 2.2.2. Image Enhancement (Pre-Processing)

During the image acquisition step, external conditions can impair the acquired image quality, such as the lighting and noise from the calibration of the cameras or the sensor. A post-process (enhancement) phase is necessary to improve the image brightness and eliminate noise (Gaussian and Impulse noise). Filtering is also necessary to improve the image quality to have better segmentation results [21,45].

#### 2.2.3. Segmentation (Processing)

The segmentation is the most delicate phase in the reconstruction process. The overall performance of the system mostly depends on it. In the blueberry image context, the regions correspond to the different holes, backgrounds, and nanoparticle structures constituting the different regions of interest. The automatic determination of the number of regions with the same characteristics (clusters) is a challenging problem [46,47].

##### 2.2.3.1. Conversion Step RGB to L*a*b

The L*a*b* space consists of a luminosity ‘L*’ or brightness-layer, chromaticity-layers ‘a*, and ‘b* indicating the color axis. For images, the information is in the chromaticity layers ‘a*, and ‘b*. The difference between the two colors was measured by using the Euclidean distance similarity metric [37,48].

##### 2.2.3.2. *K*-Means Clustering

**Figure 3:**
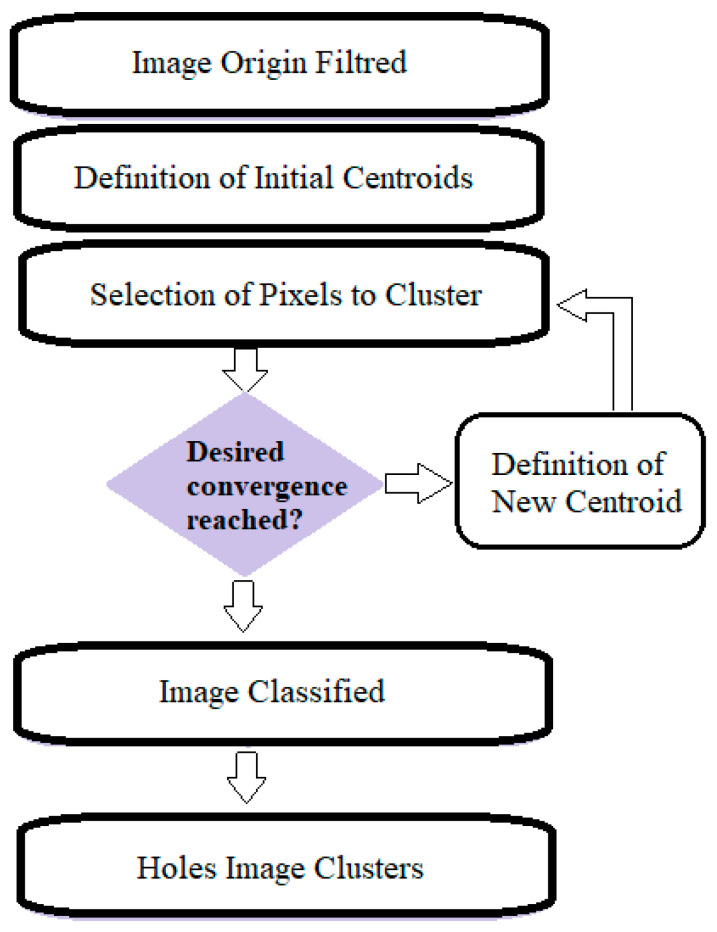
The flow chart of *K*-means clustering.

Clustering is a used method to divide data into different groups. *K*-means method is an unsupervised clustering method that classifies the input data objects into multiple classes basing on their distance [49]. An iterative calculation of Euclidean distance between the total data and the center is done. When the error becomes less than the small threshold prefixed and the maximum number of iterations was finished, the convergence was reached, Figure 3.

**Figure 4:**
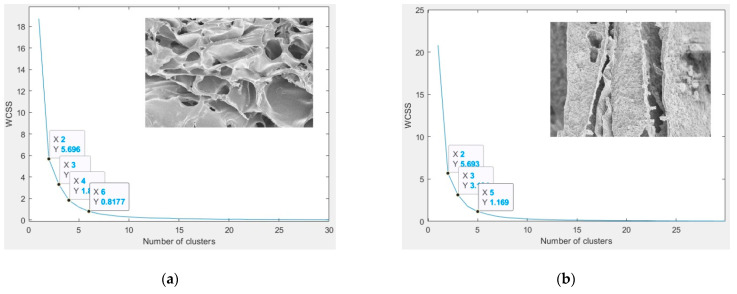

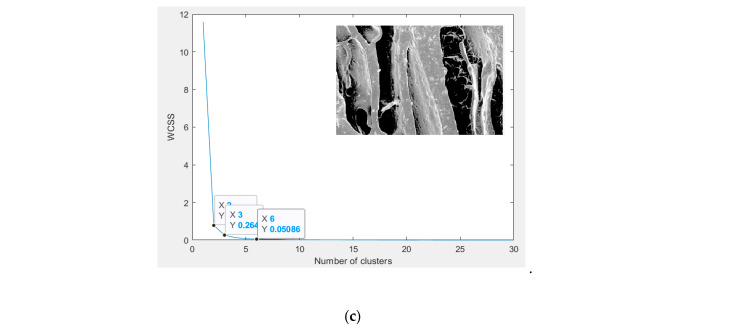
Highlighting the *K* optimal clusters number by elbow method; Chitosan 2 to 6 clusters (**a**), Chitosan/Silica Nanoparticle 2 to 5 clusters (**b**), Chitosan/Silica Nanoparticle/N 2 to 6 clusters (**c**).

To find the optimal number of clusters “*K*”, the proposed method determined the cluster number automatically by using the elbow method and the within-cluster sums squares [50]. The location of a knee in the plot is considered as an indicator of the appropriate number of clusters. Adding another cluster does not improve the partition much better. Figure 4a shows the curve of the elbow method. It appears at the total within-cluster sum of squares (WSSC) as a function of the number of clusters. The analysis of the graph shows a curvature ranging from 2 to 5 clusters. It was observed that there was a maximum of five clusters in the images, Figure 4b. The method seemed to suggest two or three clusters.

##### 2.2.3.3. Median Filter and Binarization

The algorithm processed the image of cluster 2 (holes) a median filtering stage, of 5 × 5 pixels mask, eliminates the small pixels (salt and pepper) and a binarization step using OTSU method to adapt the threshold according to the image [51] is necessary. Finally, the regions (holes) were extracted.

#### 2.2.4. Regions Characterization (Post-Processing)

The proposed approach extracted the object and texture from the images and defined the morphological parameters to characterize the three classes. Based on the extraction of the relevant parameters in the areas made it possible to establish the right classes. Nevertheless, extracting some attributes such as the number of regions (areas), the size, total area size, the perimeter, and the ratio between the holes and the background. 

### 2.3. Statistical Analysis

A comparison of the standard means (SD±) between films was performed by using ANOVA, SPSS, and Tukey’s Post Hoc tests for the physical, mechanical measurements, and the microbial contamination of blueberry fruits.

## 3. Results and Discussion

### 3.1. Physical–Chemical Characteristics 

**Figure 5:**
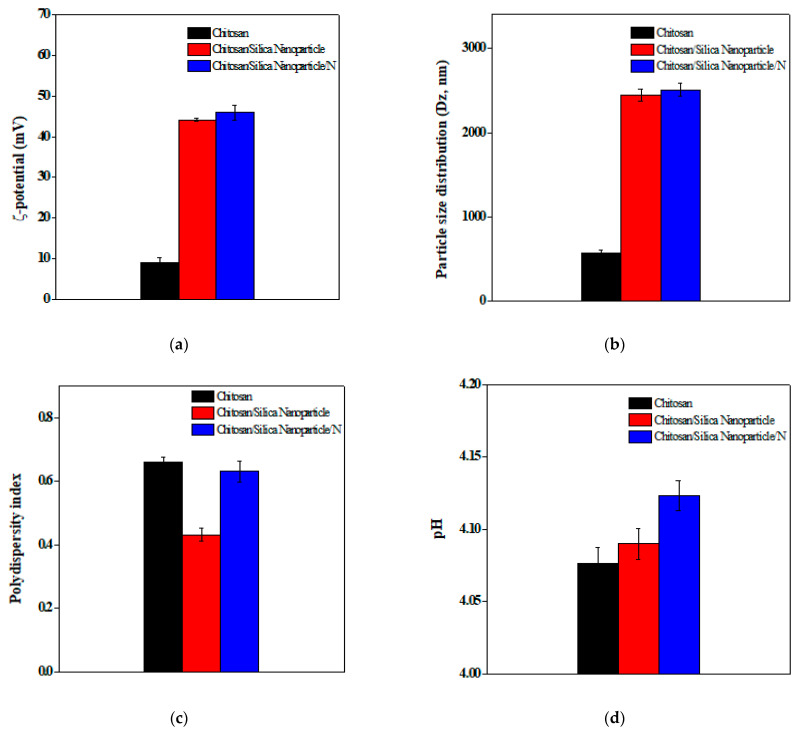

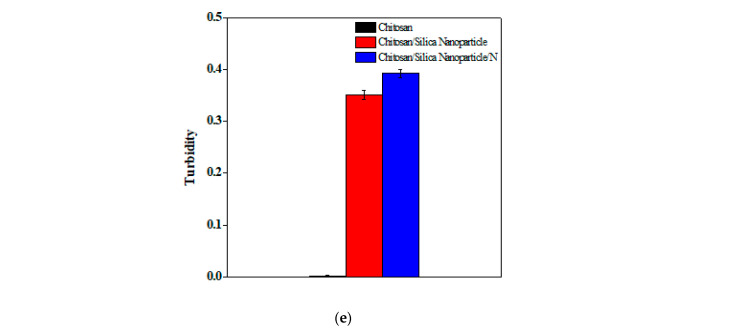
Some physical–chemical characteristics; ζ-potential (**a**), particle size distribution (**b**), polydispersity index (**c**), acidity (**d**), and turbidity (**e**) of the novel films.

The ζ-potential, particle size distribution, polydispersity index, acidity, and turbidity of the novel films are presented in Figure 5. ζ-potential of the novel films ranged from 8.92 mV in Chitosan to 45.9 mV in Chitosan/Silica Nanoparticle/N films, respectively, Figure 5a. Syamdidi et al., [51] reported that nanoparticles with a ζ-potential above 40 mV can present a stable suspension as the surface loads may prevent aggregations among particles. The reasonably stable ζ-potential values could be due to the presence of nisin that take a part in reducing the tension between solid–liquid surfaces and blocks the aggregations between particles. The distribution of particle size diameter range is presented in Figure 5b. The particles were with mean diameters ranged from 568.97 in Chitosan to 2506 nm in Chitosan/Silica Nanoparticle/N films, respectively. The surface binding of Silica Nanoparticle/N molecules with chitosan raised the size diameter of the nanoparticles [52]. The polydispersity index is an indicator of the molecule distributions. Polydispersity index of the novel films ranged from 0.43 in Chitosan/Silica Nanoparticle to 0.66 Chitosan films, Figure 5c. The distribution is considered broad when the polydispersity index ≥0.5, whereas the ideal formulation conditions as monodispersed for ≤0.3 values [53]. The pH among various novel films was changed according to the components in between 4.07 and 4.12. The result shows that the pH value was increased gradually with the addition of nisin to 4.12, Figure 5d. Chitosan films had the lowest turbidity that could be due to theirsufficient electrostatic and steric hindrance. On the other hand, the addition of Silica Nanoparticle 1% increased the turbidity to reach 0.35 and Chitosan/Silica Nanoparticle/N films recorded the highest at 0.39, Figure 5e.

### 3.2. Solubility in Water and Contact Angle 

**Figure 6:**
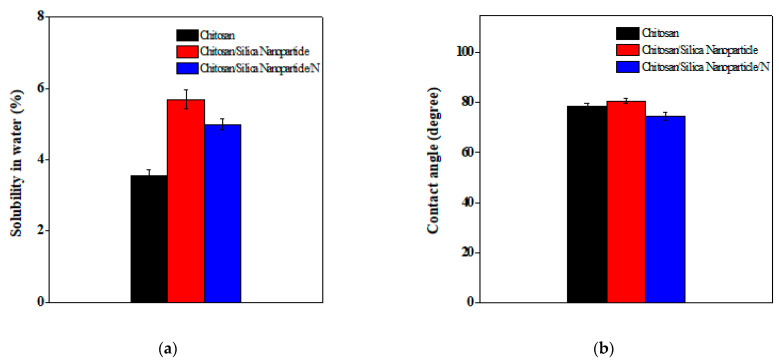
Solubility in water (**a**) and contact angle (**b**) of the novel films.

Figure 6 presents the solubility in water and contact angle of the novel films. It is clearly observed that the content of the insoluble matter was the highest for the Chitosan/Silica Nanoparticle film at 5.68%. On the other hand, Chitosan film was 3.56% and the insoluble matter decreased to 4.99% after the addition of nisin, Figure 6a. The dramatic effect of nisin on the solubility in water could be due to the presence of ionic polar, hydrophilic groups, molecules cross-linking degree which formed a denser structure and decreased the water absorption as a result [36,41].

The contact angle is needed to relate the dry weight to the hydrated weight after the filtration throughout with pre-moistened filter papers, followed by oven drying at 80 °C until reaching a constant weight. The wetting property is a vital indicator for adsorption, adhesion, and it can be influenced by the chemical compositions and material surface roughness [54]. The contact angle measurements on the novel films are presented in Figure 6b. It was observed that Chitosan/Silica Nanoparticle films have the highest degree followed by Chitosan films. On the other hand, the addition of nisin changed the contact angle to be the lowest, at 74.67 °C. Ngadiman et al. [55] reported similar results for contact angle as the insertion of nisin in Chitosan/Silica Nanoparticle films induced a change in the wettability of Chitosan/Silica Nanoparticle/N films from hydrophilicity to hydrophobicity. In the case of Chitosan films, the polar functional groups of the chitosan can restrict the hydrogen bonding interactions which increase the hydrophobicity.

### 3.3. Mechanical Properties

The mechanical properties of the novel films are presented in (Table 1). The tensile strength capacity can be influenced by the cohesive forces among intermolecular [41]. The mechanical characteristics were varied due to the various components of the novel films. Elongation-at-break of Chitosan films alone and after the addition of Silica Nanoparticle and nisin exhibited greater resistance to the strain. Llanos et al. [13] reported high values among Chitosan films in stress tests due to the porosity of the membranes. Chitosan/Silica Nanoparticle films showed a higher strain of 12.49% compared with Chitosan/Silica Nanoparticle/N films at 5.25%. As a result, the use of nanomaterials in chitosan films decreased the material ductility, reduced the tensile strength and elongation-at-break of the membrane. 

The novel films reported some structural changes in chitosan chains that had an obvious impact on the physical characteristics such as the elastic modulus for the rupture. The results reported that the membranes in Chitosan/Silica Nanoparticle exhibited an increase in elastic modulus 2233.03 MPa compared to membranes in Chitosan alone, while in Chitosan/Silica Nanoparticle/N films it recorded the lowest 569.19 MPa. Consequently, the addition of Silica Nanoparticle exhibited a ductile behavior, while the addition of nisin exhibited a fragile behavior on films. It could be due to the phenomenon of reinforcement effect of the phase separation problem “agglomeration”.

### 3.4. Microbial Contamination Analysis 

Aerobic bacteria counts increased from day 0 to day 9 along with all treatments as presented in (Table 2). Initial counts in the untreated and Chitosan samples were higher than the nano-coated blueberries. At the end of the storage period, the aerobic bacteria population increases were higher in the untreated and Chitosan samples at 4.23 and 3.90 log CFU/g, respectively. On the other hand, coated blueberries with Chitosan/Silica Nanoparticle/N films reported the lowest counts at 2.82 log CFU/g followed by Chitosan/Silica Nanoparticle at 3.73 log CFU/g. High variability of initial microbial counts is influenced by harvest conditions, fruit wetness, and the absence of natural protective wax bloom of blueberry fruits [56]. The coatings films presented antibacterial property and protective action on blueberry fruits [57,58].

Blueberries treated with Chitosan and Chitosan/Silica Nanoparticle solutions reported similar values in molds and yeasts counts during storage, Table 3. Chitosan as a main component of coating films was effective against several antimicrobial types such as fungus, molds, and yeasts counts [57]. The increase over time of molds and yeasts counts for untreated samples at 4.62 log CFU/g was the highest compared with samples coated with Chitosan/Silica Nanoparticle/N films at 3.58 log CFU/g. Results suggested that Chitosan/Silica Nanoparticle/N coating film is effective for the shelf life extension for blueberry fruits. 

### 3.5. Morphological Properties 

**Figure 7:**
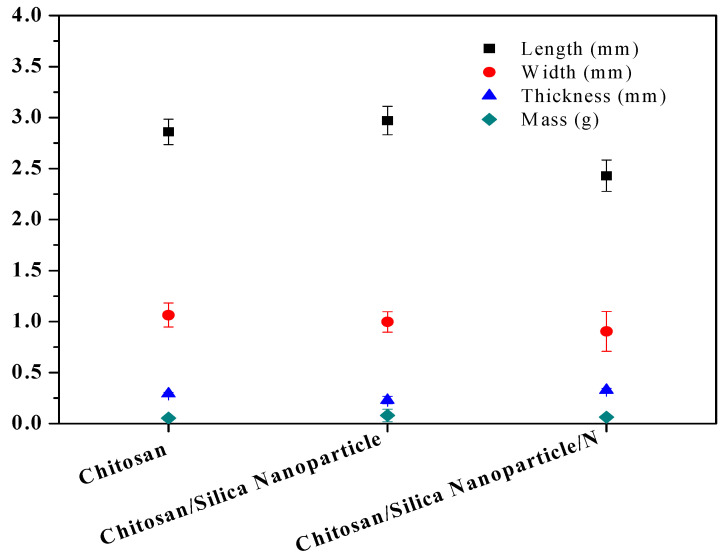
Some morphological properties of novel films.

The summary of the measured films was collated andanalyzed, andis shown in Figure 7. Length, width, and thickness values varied irregular distributions with wide ranges. Chitosan/Silica Nanoparticle established the longest film length of 2.97 mm, Chitosan reported the largest width of 1.06 mm, while Chitosan/Silica Nanoparticle/N established the shortest length and width with the largest thickness (non-plasticized and plasticized) at 0.327. Chitosan/Silica Nanoparticle/N films were thicker due to the compacting differences of the chains among the components and their interactions [58]. The dimension knowledge is essential for the aperture size of machines and separation of materials during the industry, while the thickness can greatly investigate the film properties. Chitosan/Silica Nanoparticle reported the highest mass of 0.08 g while Chitosan alone and Chitosan/Silica Nanoparticle/N films were slightly brittle. Using antimicrobial agents allows for the production of softer films and multilayer covered films [59].

### 3.6. Color Index

The color characteristics of the novel films are presented in Table 3. The lowest value of lightness was obtained for Chitosan films at 09.59. Compared to the values of the Chitosan films, a* values were decreased and b* values were increased after the addition of Chitosan/Silica Nanoparticle −2.50 ± 0.18 and Chitosan/Silica Nanoparticle/N 10.34, respectively. 

### 3.7. Image Segmentation

#### 3.7.1. Chitosan (CH) Film

The clustering algorithm assumes a vector space is formed from the data features and tries to identify natural clustering. Objects were clustered around the centroids. It is the point at which the sum of distances from all the objects in the cluster was minimized. *K*-means has the great advantage of being easy to implement. Its disadvantage is in the quality of the final clustering results which depends on the arbitrary selection of the initial centroid. The initial center must be chosen carefully to get the desired segmentation. The second parameter is the empirical choice of the *K* cluster. The algorithm allows choosing this parameter automatically according to the image.

**Figure 8:**
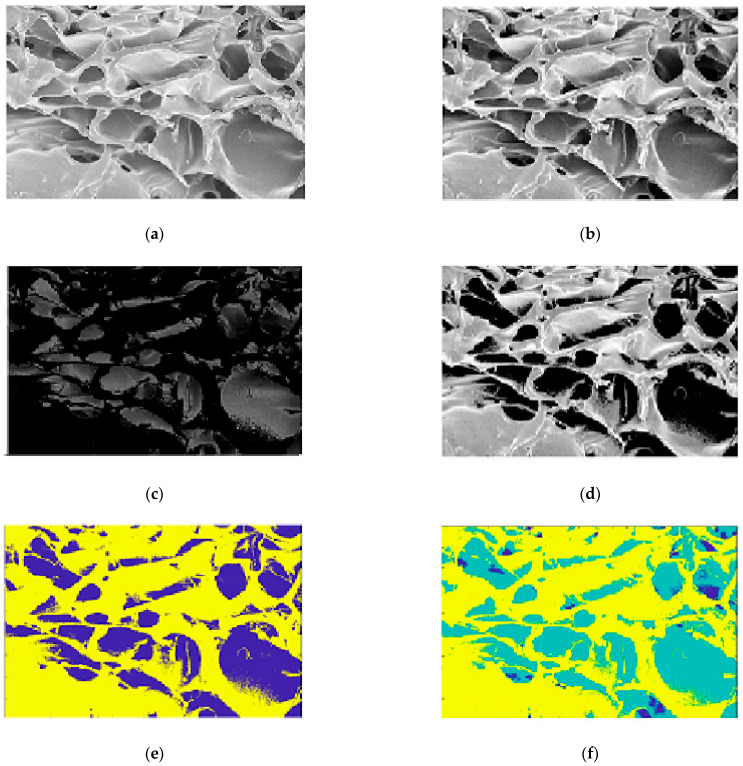
Classification: (Case1) Chitosan image origin (**a**), image enhanced (equalization) (**b**), classification cluster 1 (**c**), classification cluster 2 (**d**), *K*-means classification (2 classes) (**e**), and *K*-means classification (3 classes) (**f**).

The classification results of Chitosan images are presented in Figure 8. Using the *K-*means method, two cluster values are considered; *k* = 2 and *k* = 3, respectively as in (Figure 8e,f). The designed method uses the value of *k* = 2 to the holes from the background. *K-*means cluster algorithm generates two classes and their cluster images were implemented: image cluster 1 (Figure 8c) and image cluster 2 (Figure 8d). The image of cluster 2 highlighted the interesting areas (holes).

**Figure 9:**
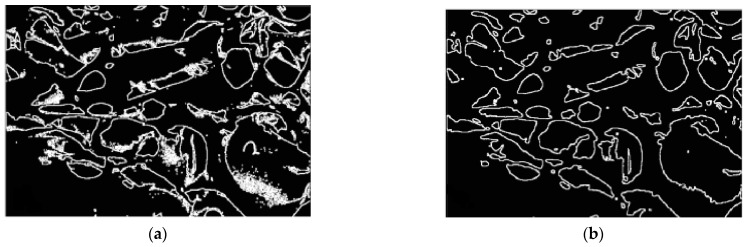
Edge extraction; (**a**) segmentation result without filter, (**b**) segmentation after median filter.

After the *K-*means classification, the proposed method generates the image of the interesting holes as in (Figure 8d). Here, the problem was the existence of some noise and isolated point which can generate false information as shown in Figure 9a. A filter step is necessary to eliminate impulse and Gaussian noise. The suggested algorithm applied a 5 × 5 median filter to deny the signals in order to improve the visual quality of the image. Figure 9b shows the result after using the median filter. 

**Figure 10:**
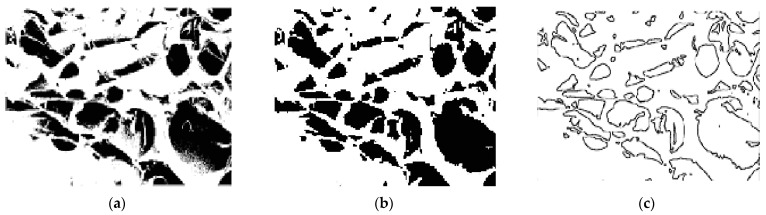
Segmentation by regions; image binarized without median filter (**a**), image filtred and binarized (**b**), and image segmented (**c**).

The results of the binarization step are shown in Figure 10a,b. Finally, an edge extraction step generates the segmentation image (Figure 10c) and all interesting areas (regions) corresponding to the holes are highlighted. The characteristics of regions areextracted well and show a good performance in quality.

#### 3.7.2. Chitosan/Silica Nanoparticle (CH-SN) Film

**Figure 11:**
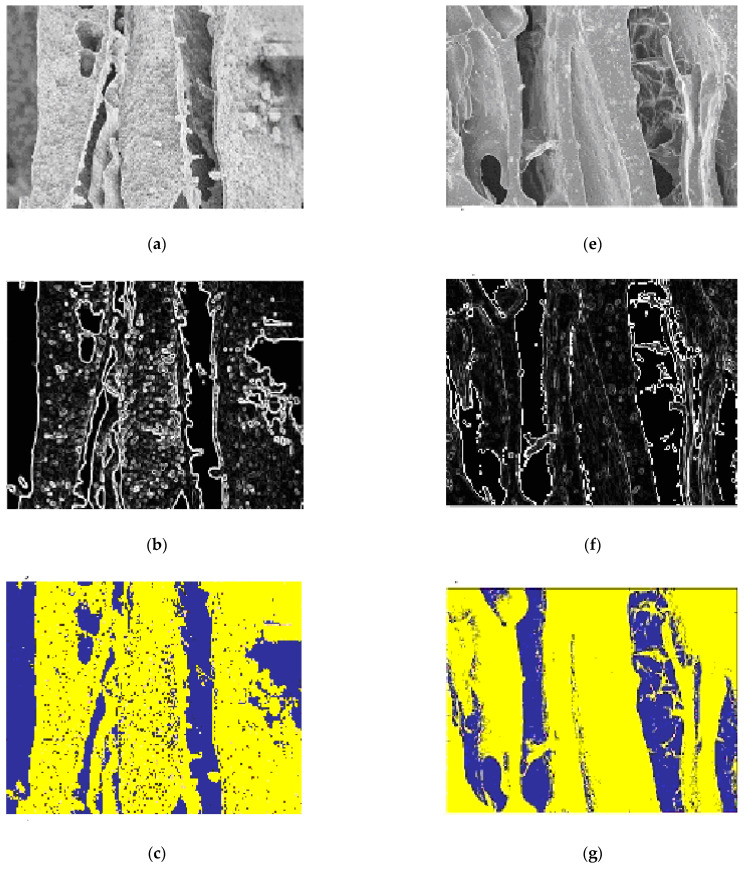

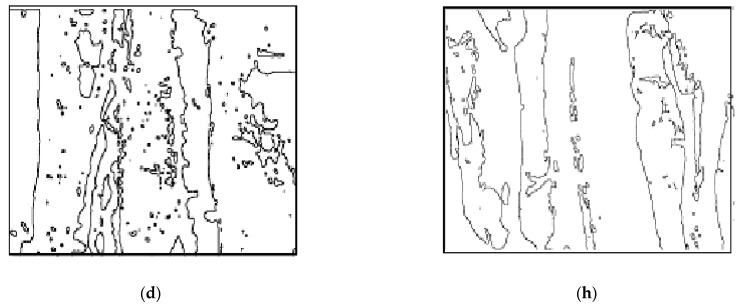
Segmentation results: (Case 2) Chitosan/Silica Nanoparticle (CH-SN); image origin (**a**), sobel edge extraction (**b**), image classified 2 classes (**c**), and image segmented (**d**). (Case 3) Chitosan/Silica Nanoparticle/Nisin (CH-SN-N); image origin (**e**), Sobel edge extraction (**f**), image classified 2 classes (**g**), and image segmented (**h**).

The algorithm was tested on Chitosan/Silica Nanoparticle (CH-SN) images (Figure 11a). The results are interesting. Figure 11b shows the result of edge extraction by the Sobel operator (3 × 3). According to the Elbow method, two clusters (with *k* = 2) are selected for the classification. Figure 11c shows the *K*-means classification results. The blue regions represent the holes and the yellow represents the background. The image of cluster 2 representing the holes is segmented by regions as shown in (Figure 11d).

#### 3.7.3. Chitosan/Silica Nanoparticle/Nisin (CH-SN-N) Film

As for the other classes, the Chitosan/Silica Nanoparticle/Nisin (CH-SN-N) images were tested by the algorithm (Figure 11e). Figure 11f shows the result of edge extraction by the Sobel operator (3 × 3). Using the Elbow method, the determined value of the *k* cluster (*k* = 2) was to classify the image in holes and background. Figure 11g shows the *K*-means classification results. The blue regions represent the holes and the yellow represents the background. The image of cluster 2 representing the holes is segmented by regions as shown in (Figure 11h).

#### 3.7.4. Characterization and Comparison of the Different Classes of Films

The regions were extracted from the image segmented. Some attributes were also extracted, such as the number of regions (areas), the size, total area size, the area’s mean, and the ratio of the holes from the total image. Table 4 summarizes the results of the characteristics of the region. 

Table 4 summarizes the characteristics results of the regions computed. The number and the size of the region extracted inform on the porosity of the film. It was observed that (CH) film extracted 94 regions with an average size of 449.10 (large size), its porosity was 9.61%; at the same time, (CH-SN) film extracted 169 regions with an average size of 130.53. This film structure was textured but the porosity is shown to be8%. The (CH-SN-N) film presented the best result at 5.19%.

#### 3.7.5. Results on Blueberry by Using the Three Classes of Films

**Figure 12:**
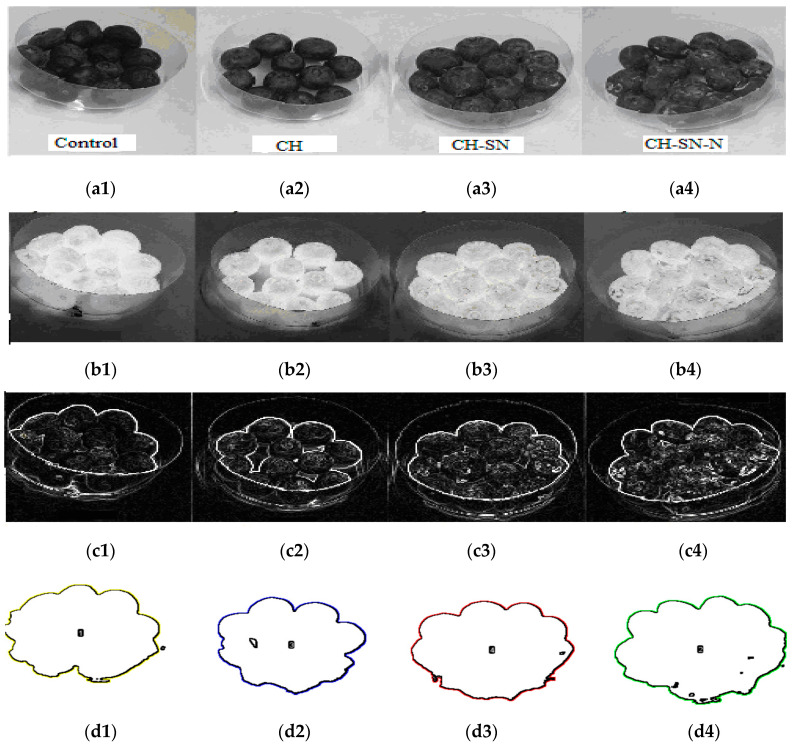
Blueberries image segmentation; H2O-Control image (**a1**), Chitosan image (CH) (**a2**), Chitosan/Silica Nanoparticle image (CH-SN) (**a3**), and Chitosan/Silica Nanoparticle/Nisin image (CH-SN-N) (**a4**). Inverse images (**b1**), (**b2**), (**b3**) and (**b4**), Sobel edge extraction (**c1**), (**c2**), (**c3**), (**c4**), and color segmented regions (**d1**), (**d2**), (**d3**), (**d4**), respectively.

Employing a good filter is needed to eliminate the noise without smoothing edges. The median filter shows that it is one of the rare filters which can de-noise an image with impulse and Gaussian noises without smoothing the image edges. Frequently, the parameter *K* (clusters) is arbitrary and it can be modified empirically. The approach aimed to define parameters automatically by analyzing the microscopic image characteristics. The analyzed images of the external structure of blueberry with films, (CH), (CH-SN), and (CH-SN-N), (Figure 12(a2, a3, and a4)), respectively, and without film (Control H2O) Figure 12(a1). Figure 12(b1, b2, b3, and b4) show the results of the inverse grey level images, which characterize the external structure of the fruit. Figure 12(c1, c2, c3, and c4) represent the extraction of contours by the Sobel method, and Figure 12(d1, d2, d3, and d4) the segmentation of the blueberry. The analysis of the obtained segmented regions and their results are grouped in (Table 5). The reference image (water control) presents a region of size of 1785 pixels. It could be observed that the size of the total region of the fruit for the (CH) case was the smallest (1663 pixels), which implied that the fruitlost moisture content. The best results were obtained for the (CH-SN-N) film as the total size of the fruit region reached 1983 pixels.

## 4. Conclusions

This study demonstrated some novel chitosan/silica nanoparticle films with the addition of nisin (CH-SN-N) as an antimicrobial technique, the characteristics of the microscopic images (SEM), and the image texture for blueberry preservation during storage. Novel nano-coatings are eco-friendly and may be efficiently used for maintaining numerous quality parameters in the research of nanotechnology applications. The (CH-SN-N) coating film presented the best characteristics and it is recommended for the reduction of molds and yeasts, aerobic bacteria plate microorganism counts in blueberry preservation.

## Figures and Tables

**Table 1:** Mechanical properties of the novel films.

Films	Elongation-at -Break	Tensile Strength, σ	Elastic Modulus, E	Breaking Force, BF	Fracture Stress, σ_F_	Extensibility
	%	MPa	MPa	gf	MPa	mm
**Chitosan**	38.64 ± 2.95 ^a^	16.53 ± 0.66 ^a^	2034.52 ± 215.83 ^a^	600.85 ± 17.71 ^a^	18.92 ± 0.84 ^a^	7.86 ± 0.49 ^a^
**Chitosan/Silica Nanoparticle**	12.49 ± 1.25 ^b^	7.65 ± 0.41 ^b^	2233.03 ± 257.58 ^a^	194.73 ± 12.69 ^b^	11.85 ± 0.90 ^a^	3.75 ± 0.57 ^b^
**Chitosan/Silica Nanoparticle/N**	5.25 ± 1.80 ^b^	0.90 ± 0.30 ^b^	569.19 ± 107.07 ^b^	27.78 ± 1.30 ^c^	0.94 ± 0.31 ^b^	1.57 ± 0.54 ^b^

Values within a column (lowercase) a;b;c are significantly different (*p* ≥ 0.05). The values in parentheses indicate (SD±) standard deviation.

**Table 2:** Effect of coating films on microbial contamination analysis of blueberry fruits during storage.

Days	Untreated	Chitosan	Chitosan/Silica Nanoparticle	Chitosan/Silica Nanoparticle/N
**Aerobic bacteria counts (log CFU/g)**				
0	1.767 ± 0.049 ^a^	1.700 ± 0.013 ^a^	1.500 ± 0.035 ^b^	1.033 ± 0.015 ^c^
3	2.200 ± 0.023 ^a^	2.033 ± 0.072 ^b^	1.900 ± 0.059 ^c^	1.367 ± 0.024 ^d^
6	3.000 ± 0.045 ^a^	2.633 ± 0.027 ^c^	2.867 ± 0.032 ^b^	1.933 ± 0.025 ^d^
9	4.227 ± 0.038 ^a^	3.901 ± 0.053 ^b^	3.733 ± 0.019 ^c^	2.823 ± 0.079 ^d^
**Molds and yeasts counts (log CFU/g)**				
0	2.033 ± 0.049 ^a^	1.900 ± 0.011 ^b^	1.867 ± 0.031 ^b^	1.800 ± 0.010 ^c^
3	2.433 ± 0.012 ^a^	2.267 ± 0.093 ^b^	2.400 ± 0.051 ^a^	2.067 ± 0.039 ^c^
6	3.200 ± 0.050 ^a^	3.067 ± 0.029 ^b^	3.267 ± 0.034 ^a^	2.467 ± 0.026 ^c^
9	4.622 ± 0.034 ^a^	4.090 ± 0.055 ^a^	4.000 ± 0.010 ^a^	3.580 ± 0.025 ^b^

Values within a column (lowercase) a;b;c;d are significantly different (*p* ≥ 0.05). The values in parentheses indicate (SD±) standard deviation.

**Table 3:** Color index of coating films.

Films	Color Index		
	L*	a*	b*
**Chitosan**	09.59 ± 0.05 ^c^	−1.90 ± 1.29 ^ab^	2.54 ± 0.49 ^b^
**Chitosan/Silica Nanoparticle**	49.39 ± 0.26 ^b^	−2.50 ± 0.18 ^b^	−1.78 ± 0.06 ^c^
**Chitosan/Silica Nanoparticle/N**	58.24 ± 0.41 ^a^	−0.67 ± 0.12 ^a^	10.34 ± 0.32 ^a^

Values within a column (lowercase) a;b;c are significantly different (*p* ≥ 0.05). The values in parentheses indicate (SD±) standard deviation.

**Table 4:** Comparison of the morphological characteristics.

Films	Region Number	Total Area	% Area	Average Area Size
**CH**	94	42,211	9.61	449.10
**CH-SN**	169	22,060	8	130.53
**CH-SN-N**	79	23,016	5.19	291.34

**Table 5:** Morphological characteristics of blueberry’s regions.

Case	Area/Pixels	Perimeter	Edge/Number of Point	Segmentation/Color Edge
**Control (H2O)**	1785	715.2	514	Yellow
**CH**	1663	663	474	Blue
**CH-SN**	1803	707.8	500	Red
**CH-SN-N**	1983	760	518	Green

## Data Availability

Available upon request from the corresponding author.
